# Myofunctional Responses in Obstructive Sleep Apnea Syndrome in Children Following the Use of Two Oral Orthopedic Devices

**DOI:** 10.4317/jced.62603

**Published:** 2025-04-01

**Authors:** Ana Beatriz Bueno Carlini Bittencourt, Clóvis Lamartine de Moraes Melo-Neto, Gabriela Aparecida dos Santos, Emily Vivianne Freitas da Silva, Claúdia Sanae Akita Shimoide Muraoka, André Pinheiro de Magalhães Bertoz, Daniela Micheline dos Santos, Marcelo Coelho Goiato

**Affiliations:** 1 Department of Dental Materials and Prosthodontics, Araçatuba Dental School, UNESP – São Paulo University, São Paulo, Brazil; 2 Department of Prosthodontics, Faculty of Dentistry – University of São Paulo (USP), São Paulo, Brazil

## Abstract

**Background:**

Rapid maxillary expansion (RME) is less invasive and an efficient method of treatment for obstructive sleep apnea syndrome (OSAS). Objective: To assess the therapeutic impact of oral orthopedic appliances in the treatment of obstructive sleep apnea syndrome with polysomnography (PSG), electromyography (EMG), bite force measurement (BFM), questionnaires, and cephalometric analysis.

**Material and Methods:**

Eleven children (aged seven to eleven years) underwent three months of treatment with the Hyrax device, followed by the installation of the Balter´s Bionator appliance. Quality analysis, type III polysomnography (PSG), electromyography (EMG), and bite force measurement (BFM) were conducted. The analysis were performed through eleven months of treatment. The distributional normality were verified by the Kolmogorov–Smirnov, For these data, the Student test was performed. For data with non-normal distribution, the Wilcoxon test was performed. All analyzes were performed with a significance level of 5%.

**Results:**

Polysomnography data showed that severe scores became moderate or mild with the use of the devices. Electromyography, during rest periods, demonstrated that only the right temporal muscle exhibited increased electrical activity, while both muscles showed increased activity during dental clenching. There was an increase in the masseter and right temporalis during grape chewing. Bite Force Measurement (BFM) did not reveal any statistical difference before and after treatment. Questionnaire responses showed a statistically significant difference in the Sleep Disturbance Scale and OSA-18-PV.

**Conclusions:**

It can be concluded that the use of the Balters Hyrax and Bionator devices in class II children and children with OSAS is a safe and effective treatment alternative.

** Key words:**Sleep apnea obstructive, bite force, polysomnography, electromyography, oral health, children.

## Introduction

Obstructive Sleep Apnea Syndrome (OSAS) is a condition that primarily affects children, characterized by repeated episodes of partial or complete obstruction of the upper airways during sleep, leading to interruptions in sleep and decrease in oxygen saturation (1,2). This syndrome is associated with obesity, medication therapies, hormonal treatments, craniofacial abnormalities, and hereditary factors (3,4). Another commonly associated factor is adenotonsillar hypertrophy, which may lead to mouth breathing, excessive daytime sleepiness, snoring during sleep, episodes of apnea, restless sleep, and coughing (5,6).

It is a multifactorial disorder that requires multidisciplinary collaboration from health professionals, including sleep specialists, pediatricians, otolaryngologists, speech therapists, and dental surgeons (7). The diagnosis is based on clinical examinations, imaging and polysomnography – considered a gold standard method - to guide the professional towards an appropriate treatment (1,4,5).

The first-line and least invasive treatment involves the use of functional orthopedic devices such as rapid maxillary expanders, Bionator, and occlusal splints (8,9). These appliances significantly reduce pauses in breathing, expand the maxillary bones, improve lip sealing, increase vertical dimension, and harmonize maxillo-mandibular relationships, as well as reposition the tongue and musculature, which helps enhance airflow and prevent upper airway collapse (10,11). Their disadvantages include initial discomfort during adaptation, a sensation of reduced saliva, communication difficulties, and reports of headaches (5,12,13).

Previous studies have shown that maxillary expansion or mandibular advancement appliances can be effective in treating OSAS (14,15). Therefore, the objective of this study was to assess the main therapeutic effects of oral orthopedic devices before and after eleven months of treatment. Analyses included polysomnography, electrical activity of the masseter and temporal muscles (providing data on muscle activity intensity levels), maximum bite force (directly influencing chewing capacity), quality of life questionnaires, and the diameter of cephalometric points NFA-NFP and BFA-BFP.

The null hypothesis of this study was that there was no significant differences in the outcomes of RME treatments (Hyrax and Bionator appliances); no difference in the electrical activity of the masseter and temporal muscles, in maximum bite force, in participants’ quality of life, and no increase in the diameter of cephalometric points.

## Material and Methods

1. Study Design and Setting

A study to analyze the therapeutic outcomes of oral orthopedic devices in children with obstructive sleep apnea syndrome through polysomnography, electromyography of the masseter and temporal muscles, maximum bite force, quality of life questionnaires, and cephalometric analyses. Data were collected before and after treatment.

2. Participants selection

The research project was submitted and approved by the Ethics Committee of the Faculty of Dentistry of Araçatuba – UNESP (Opinion 3.401.309) and is registered in the Plataforma Brasil (CAAE: 13909119.0.0000.5420).

Eleven children aged between seven and eleven years, of both genders, were selected from an Educational Center in the city of Araçatuba / SP. Then, at the Faculty of Dentistry of Araçatuba – UNESP, initial parameters for diagnosis and therapeutic analysis were conducted. All information related to the intervention was explained to the parents, who consented and signed the Free and Informed Consent Term for children (TCLE).

3. Inclusion and exclusion criteria

The study subjects were patients who met the following inclusion criteria: symptoms of OSA (restless sleep, excessive daytime sleepiness, class II malocclusion, irritability); good general health; maxillary and mandibular bone support; intact upper and lower permanent posterior teeth. Children were excluded if they had any of the following conditions: neurological disorders, dental prosthetics or orthodontic appliances, history of facial trauma, mandibular advancement limitation less than 5mm, prevalence of dental pain, temporomandibular dysfunction (TMD), speech therapy treatment, otolaryngological treatment, use of psychotropic or muscle relaxant medications.

4. Clinical Sequence

4.1 Initial Diagnosis and Analysis

After clinical examination, Type III PSG variables were scored to diagnose OSAS and radiographs with cephalometric tracings (rest position, maximum mandibular protrusion, and NFA-NFP/BFA-BFP points) were analyzed to confirm class II malocclusion. Parents of participants completed the Sleep Disturbance Scale for Children and OSA-18-PV questionnaire.

To diagnose OSAS, participants underwent Type III PSG (Type III porTable monitoring device Stardust II® - Philips Respironics). The Stardust records include: airflow (nasal pressure), heart rate (finger sensor), respiratory effort (belt with piezoelectric sensor set at the lower sternum and body position (device positioned at the lower sternum).

EMG was performed using a surface electromyography device brand DataHominis MyosystemBs1 model to assess masseter and temporal muscles activity. Prior to the exam, participants underwent hygiene procedures (water, soap, and 70% alcohol). Afterwards, in sitting position, with their hands on their laps and head positioned according to the Frankfurt plane, the analysis was conducted by a single examiner. Data were recorded in mandibular rest position (five seconds); in maximum intercuspation (MIH) with and without Parafilm M tape (Bemis Flexible Packaging, Neenah, WI, USA) (five seconds); and chewing of 3g of raisins and shelled peanuts (ten seconds) (16,17).

The maximal molar bite force (MBF) was assessed using a digital dynamometer, model IDDK (Kratos - Equipamentos Industriais Ltda., Cotia, São Paulo, Brazil), fitted with two sticks (protected with disposable latex finger cots - Wariper-SP), to which the bite force was applied. The measurements were alternately taken in the region of the first right and left molar teeth on each side of the dental arch, and incisors (16,18).

4.2 Rapid maxillary expansion (RME) 

The first device fabricated was Hyrax. Pick-up impressions were taken with alginate and poured with orthodontic plaster. On stone cast, screw was installed. A strip of wax was placed on the palate to allow distance from the screw, and its installation was centered in the region of the deciduous premolars and molars. Parents were instructed to follow a protocol of activation of a quarter turn in the morning and a quarter turn at night for two weeks. The appliance was kept in situ as retention for three months.

Afterwards, the device was removed and Balter´s Bionator was fabricated following the same steps to obtain the stone cast. The Bionator appliance consisted of an acrylic monobloc (Colorless Classic, JET) with a vestibular guiding arch, a palatal bar (coffin spring) and a buccinator loop, covering the occlusal surface of the posterior teeth (19,20). Participants were instructed on hygiene, maintenance, and subsequent follow-ups, returning twice a month at the Faculty of Dentistry of Araçatuba clinic.

**Figure 1:**
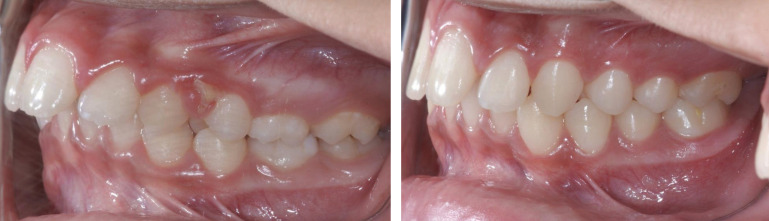
Measurements of cephalometric points before and after treatment with ERM.

As soon as the treatment for OSAS was completed, patients returned to the clinic for a new evaluation: Type III PSG exams, EMG of the masseter and temporal muscles, maximum bite force after the device was removed, questionnaires, and final orthodontic documentation, (Fig. 1).

5. Statistical analysis

Statistical analysis was performed using the SPSS version 24.0 software (SPSS Inc., Chicago, IL, USA). The normal distribution of the data was assessed with the Kolmogorov-Smirnov Z-test to dental clenching measurements with parafilm M. The following data were observed: records from polysomnography; electromyography of the right masseter (except at rest position); electromyography of the left masseter (except without parafilm and grape); electromyography of the right temporal muscle; electromyography of the left temporal muscle (except at rest position, without Parafilm, and peanut); bite force in the incisor and left / right molar regions; Sleep Disorder Scale and OSA-18-PV questionnaire data; and cephalometric NFA-NFP and BFA-BFP points. For these data, Student paired T test was used for continuous matched pairs of normal data and the Wilcoxon signed rank test for nonparametric variables. All analyses were performed with a significance level of 5%.

## Results

All the changes induced by RME on the upper jaw and adjacent structures were analyzed by electrical activity of the masseter and temporal muscles, maximum molar bite force, and cephalometric evaluation.

Recordings from the polysomnography examination ( Table 1) observed that there was a statistically significant difference after the use of the orthopedic devices. At the beginning of the study, a percentage of 27.3% identified as having a “severe” score changed to “moderate”, after RME treatment. Patients identified as “mild” score showed a significant increase.

Data from the electromyographic evaluation at clinical condition of rest ( Table 2) showed a significant increase in electrical activity (*p* = 0.041) only in the right temporal muscle. During dental clenching without parafilm ( Table 2) there was no statistically significant differences, however it was noted an increased activity in both muscles.

There were no statistically significant differences in masticatory function for habitual chewing with peanuts ( Table 2). There was an increase in electrical activity for habitual chewing with raisins and statistically significant differences were observed in the right masseter (*p* = 0.003) and temporal (*p* = 0.042) muscles after the use of the orthopedic devices. There was no statistical difference for maximum bite force in permanent posterior teeth and incisors ( Table 3).

According to questionnaire responses ( Table 4), there was significant statistical difference for sleep disorder scale (*p* = 0.007) and in the OSA-18-PV (*p* = 0.010). Values in the assessment of the cephalometric points are shown in  Table 5, and a statistical difference was observed at the NFA-NFP point (*p* = 0.043).

## Discussion

The first null hypothesis - no significant differences in the outcomes of RME treatments - was rejected, as a positive change in scores and a reduction in PSG values were observed before and after treatment. The second null hypothesis - no difference in the electrical activity of the masseter and temporal muscles - was also rejected, as there was an increase in electrical activity at clinical condition of rest for the right temporal muscle and for habitual chewing with raisins for both muscles.

The third null hypothesis - no difference in maximum bite force (MBF) - was accepted, as the values remained sTable in both periods. The fourth null hypothesis - no difference in the quality of life of the participants - was rejected, since the data obtained from questionnaires showed an improvement in sleep disorders, according to the parents’ perceptions. The fifth null hypothesis - no increase in the diameter of the cephalometric points - was rejected, as there was a significant difference in the linear distance between NFA-NFP points (anterior and posterior nasopharynx).

Statistical differences shown in  Table 1 reinforce that both Balter´s Bionator and Hyrax appliances are beneficial for patients with obstructive sleep apnea (21). The Bionator has a mechanism that promotes an increase in vertical growth of the ramus and stimulate forward mandibular growth, improving the maxillomandibular relationship with an adequate control of the position of the lower incisors (22).

Additionally, therapeutic mandibular advancement as a treatment option for Angle Class II increases airflow, reducing symptoms of OSAS. The Hyrax device improves not only bone dimensional level but also respiratory function (23). These promising findings suggest that RME devices should have a useful role in the therapy of OSAS in children (24).

The consequences of sleep problems can vary from daytime sleepiness to headaches, behavioral problems, poor school results (25,26). The present study demonstrated that RME therapy improved sleep quality and general well-being. Similarly, Capalbo *et al*., (27) showed that soon after RME, apnea and hypopnea index decreased.

The position of the temporal muscle and occlusal changes through growth stages may have required greater EMG activity from the muscle on one side (right side —  Table 2). Once occlusion is stabilized, there is a reduction in electrical activity (28). According to Miyata (29), it is suggested that chewing efficiency depends on dental growth and development.

The most important muscles of mastication are the temporal and the masseter, responsible for vertical, lateral and antero-posterior movement of the mandible (30). Therefore, considering that orthopedic devices can influence their function, these muscles were evaluated before and after treatment with RME, correlating their effects with improvements in obstructive sleep apnea syndrome (OSAS) in the individuals.

In  Table 3, there is a variability in the results for bite force due to mandibular positioning, stage of dentition, dental sensitivity, physical activity, and fear of damaging teeth during the tests (31). Cephalometric (NFA-NFP distance) data showed an increase in the upper airway space ( Table 5). Many authors demonstrated that mandibular advancement devices and rapid maxillary expanders can stabilize the pressure along the pharynx, preventing obstructions during sleep (23,32).

An unavoidable limitation of our study is that the number of patients that completed the treatment were reduced due to the COVID-19 pandemic era. Additionally, psychological factors in patients through dental transition may have influenced the bite force measurements exam.

Our open trial suggests that dentists should consistently look for dental anomalies in children and inquire with parents about any chronic snoring or other symptoms of obstructive sleep apnea (OSA). Furthermore, the trial indicates that orthodontic treatment using rapid maxillary expansion (RME) can provide significant benefits for children with OSA. These encouraging findings highlight the need for controlled studies to validate the effectiveness of RME in the treatment of obstructive sleep apnea syndrome (OSAS).

## Conclusions

The use of the Hyrax and Balter´s Bionator appliances in class II children with Obstructive Sleep Apnea Syndrome is a safe and effective treatment, as there was no damage to bite force and muscle electrical activity.

RME treatment has a positive effect on children affected by chronic snoring and OSA. By changing the anatomic structures, RME brings a functional improvement.

## Figures and Tables

**Table 1:** Polysomnography results at baseline and after 11 months of treatment.

Polysomnography	Initial	Final	p value
Value	5,69 ± 1,27	3,85 ± 1,69	0,020*^†^
Score	Mild: 18,2% Moderate: 54,5% Severe: 27,3%	Mild: 81,8% Moderate: 18,2%	0,008*^‡^

*Significant values at *p*<0,05 ate reported. Student T-test; ‡Wilcoxon Test.

**Table 2:** Mean and standard deviations of the electromiograpy (μV) from masseter and temporal muscles at clinical condition of rest; dental clenching withou parafilm; chewing peanuts; and chewing grapes.

Muscle	Initial: EMG activity DC without parafilm C peanut C grape	Final: EMG activity DC without parafilm C peanut C grape	p value: EMG activity DC without parafilm C peanut C grape
Right Masseter	0,03 ± 0,06 0,98 ± 0,27 0,55 ± 0,16 0,35 ± 0,15	0,03 ± 0,03 1,17± 0,39 0,69 ± 0,52 0,59 ± 0,26	0,110^‡^ 0,086^†^ 0,319^†^ 0,003^*†^
Left Masseter	0,01 ± 0,01 1,03 ± 0,31 0,58 ± 0,14 0,42 ± 0,14	0,02 ± 0,02 1,04 ± 0,24 0,59 ± 0,35 0,54 ± 0,26	0,113^†^ 0,424^‡^ 0,981^†^ 0,328^‡^
Right Temporal	0,03 ± 0,03 1,04 ± 0,29 0,61 ± 0,19 0,37 ± 0,15	0,07 ± 0,05 1,14 ± 0,19 0,54 ± 0,20 0,48 ± 0,12	0,041^*†^ 0,230^†^ 0,258^†^ 0,042^*†^
Left Temporal	0,04 ± 0,05 1,08 ± 0,35 0,64 ± 0,13 0,46 ± 0,15	0,09 ± 0,11 1,15 ± 0,26 0,79 ± 0,43 0,56 ± 0,15	0,062^‡^ 0,182^‡^ 0,328^‡^ 0,084^†^

*. *Significant values at *p*<0,05 ate reported. Student T-test; ‡Wilcoxon Test. DC: dental clenching; C: chewing.

**Table 3:** Mean and standard deviations of the bite force (kg / F).

Bite force	Initial	Final	p value
Right molar	248,53 ± 101,90	246,49 ± 131,36	0,722^‡^
Left molar	251,94 ± 93,35	241,75 ± 100,91	0,738^†^
Incisive	62,97 ± 43,09	73,86 ± 30,06	0,248^‡^

*Significant values at *p*<0,05 ate reported. Student T-test; ‡Wilcoxon Test

**Table 4:** Mean and standard deviations of the sleep disturbance scale; answers to Questionnaire from 0 to 10.

Bite force	Initial	Final	p value
Sleep disturbance scale	56,54 ± 12,01	42,91 ± 8,60	0,007^*^
OSA-18 PV	7,54 ± 11,51	8,72 ± 1,42	0,010^*^

*Significant values at *p*<0,05 ate reported. Student T-test; ‡Wilcoxon Test

**Table 5:** Mean and standard deviations of the cephalometric measurements.

Bite force	Initial	Final	p value
NFA-NFP	8,82 ± 3,85	11,40 ± 2,78	0,043^*^
BFA-BFP	10,95 ± 2,28	10,59 ± 1,65	0,362

*Significant values at *p*<0,05 ate reported. Student T-test; ‡Wilcoxon Test

## Data Availability

The data that support the findings of this study are available on request from the corresponding author. The data are not publicly available due to privacy or ethical restrictions.
